# Natural and experimental hepatitis E virus genotype 3 - infection in European wild boar is transmissible to domestic pigs

**DOI:** 10.1186/s13567-014-0121-8

**Published:** 2014-11-26

**Authors:** Josephine Schlosser, Martin Eiden, Ariel Vina-Rodriguez, Christine Fast, Paul Dremsek, Elke Lange, Rainer G Ulrich, Martin H Groschup

**Affiliations:** Institute for Novel and Emerging Infectious Diseases, Friedrich-Loeffler-Institut, Südufer 10, 17493 Greifswald-Insel Riems, Germany; Department of Experimental Animal Facilities and Biorisk Management, Friedrich-Loeffler-Institut, Südufer 10, 17493 Greifswald-Insel Riems, Germany

## Abstract

Hepatitis E virus (HEV) is the causative agent of acute hepatitis E in humans in developing countries, but sporadic and autochthonous cases do also occur in industrialised countries. In Europe, food-borne zoonotic transmission of genotype 3 (gt3) has been associated with domestic pig and wild boar. However, little is known about the course of HEV infection in European wild boar and their role in HEV transmission to domestic pigs. To investigate the transmissibility and pathogenesis of wild boar-derived HEVgt3, we inoculated four wild boar and four miniature pigs intravenously. Using quantitative real-time RT-PCR viral RNA was detected in serum, faeces and in liver, spleen and lymph nodes. The antibody response evolved after fourteen days post inoculation. Histopathological findings included mild to moderate lymphoplasmacytic hepatitis which was more prominent in wild boar than in miniature pigs. By immunohistochemical methods, viral antigens were detected mainly in Kupffer cells and liver sinusoidal endothelial cells, partially associated with hepatic lesions, but also in spleen and lymph nodes. While clinical symptoms were subtle and gross pathology was inconspicuous, increased liver enzyme levels in serum indicated hepatocellular injury. As the faecal-oral route is supposed to be the most likely transmission route, we included four contact animals to prove horizontal transmission. Interestingly, HEVgt3-infection was also detected in wild boar and miniature pigs kept in contact to intravenously inoculated wild boar. Given the high virus loads and long duration of viral shedding, wild boar has to be considered as an important HEV reservoir and transmission host in Europe.

**Electronic supplementary material:**

The online version of this article (doi:10.1186/s13567-014-0121-8) contains supplementary material, which is available to authorized users.

## Introduction

Hepatitis E virus (HEV) is the causative agent of hepatitis E in humans and the sole member of the genus *Hepevirus* in the family *Hepeviridae*. It is a small, non-enveloped virus with a single-stranded RNA genome of positive polarity [1,2]. In many developing countries where sanitary conditions are suboptimal, hepatitis E is an important public health problem, with the virus being primarily transmitted via the fecal-oral route through contaminated food or water [3]. However, emerging cases of sporadic and autochthonous hepatitis E also occur in industrialised countries, including Japan and European countries [4-6]. HEV infections are known to be responsible for acute hepatitis, however, HEV genotype 3 was recently also identified in Europe in severely immunocompromised patients as a new causative agent of chronic hepatitis [7,8]. Four genotypes (gt) of HEV (gt1 to gt4) infecting humans have been identified. Gt1 and gt2 are restricted to humans, and gt3 and gt4 are zoonotic with wild boar, domestic pig and deer representing reservoirs [9,10]. Although a consensus classification system for HEV genotypes is currently unavailable, HEV variants from Japanese wild boar (*Scrofa scrofa leucomystax*) have provisionally been classified into two novel genotypes (gt5 and gt6) [11]. The identification and characterization of additional HEV strains in chicken, rabbit, different rat species, and mongoose have significantly broadened the host range and diversity of HEV [12,13]. Recently, novel HEV-related viruses were identified in carnivores such as ferret [14] and fox [15], different bat species [16], moose [17] and cutthroat trout [18]. Cross-species transmission to non-human primates and pigs have been shown experimentally for gt3 and gt4 [19]. Severe human HEV infection after ingestion of uncooked liver from wild boar *S. scrofa leucomystax*) was reported in Japan, whereas food -borne zoonotic transmissions in Europe have been primarily associated with domestic pigs [4,20]. Furthermore, individuals with direct contact to pigs are at higher risk of HEV infection and as previously shown, forestry workers have a higher HEV seroprevalence rate compared to blood donors [21-23]. Recent studies in Asia and Europe revealed high HEV seroprevalences and molecular evidence for HEV infection in wild boar [24-31]. In Germany, wild boar is discussed as one of the main sources of human autochthonous infections [32,33]. Moreover, phylogenetic analyses of Japanese HEV isolates indicated former transmission events from domestic pig to wild boar [34].

Until now several studies in domestic pigs have been performed by intravenous or contact transmission of domestic pig-derived HEV [35-40], showing histopathological signs of a hepatitis but no clinical symptoms [37,41,42]. Conversely, little is known about the course of HEV infection in European wild boar and their role in HEV transmission to domestic pigs to date. Experimental challenge studies have not been carried out yet. Therefore, the aim of this study was to investigate the pathogenesis of a wild boar-derived HEV gt3 strain after experimental inoculation and to reveal possible horizontal transmissions to miniature pigs (*S. scrofa domestica*) and European wild boar (*S. scrofa scrofa*).

## Materials and methods

### Inoculum

The HEV gt3 strain used in this study originated from a liver sample of a naturally infected wild boar hunted in Northern Germany (Mecklenburg-Western Pomerania) in 2010. The liver was frozen immediately at −20 °C and stored at −70 °C. For preparation of the inoculum, the liver was ground in phosphate-buffered saline (PBS) with a mortar and pestle (10%, w/v). The suspension was transferred to a 15 mL tube and mixed for 1 min using a vortex mixer. After centrifugation (20 min at 4000 × *g* at 4 °C) the supernatant was transferred to a new tube and filtered (0.22 μm MILLEX®GP filter unit, Millipore, Ireland). The suspension was aliquoted in volumes of 2.5 mL and stored at −70 °C. The inoculum contained about 2 × 10^4^ HEV RNA copies per μL RNA.

### Experimental design

Seven sub-adult miniature pigs of three months age, three wild boar piglets of three months age and two adult wild boar of six month age were used in the experiment under biosafety level 3** conditions. Prior to the start of the experiment all animals were tested to be negative for anti-HEV antibodies in serum and HEV RNA in faeces, respectively. The wild boar piglets used in the study were obtained from a local farmer. Miniature pigs and adult wild boar were bred in the quarantine facilities at the Friedrich-Loeffler-Institut, Insel Riems, Germany. Following an initial clinical examination, including rectal body temperature, wild boar were allowed to accustom themselves to new surroundings for approximately 1–2 weeks prior to the initiation of experiments. The animals were fed with commercial pig feed and had access to water *ad libitum*. Two additional miniature pigs and one additional wild boar served as negative controls and were housed separately. Control animals remained negative for the whole experiment and were not considered further on in this manuscript. All animals were observed daily during the entire period of the experiment. In Group 1 and Group 2, four wild boar (wb93, wb95, wb10 and wb11) and four miniature pigs (mp30, mp37, mp39 and mp40) were inoculated intravenously via the *vena cava cranialis* with 2.0 mL liver suspension each. For the direct contact infection experiment (Group 3), one non-inoculated wild boar piglet (wb87) was kept together with the intravenously inoculated wild boar piglets (wb93 and wb95). For animal welfare reasons three miniature pigs (mp63, mp68 and mp79) were kept in an adjacent compartment. To facilitate an indirect transmission, excrements of intravenously inoculated wild boar (wb93 and wb95) were placed daily into stable of miniature pigs. Conveniently, time points of the experiment were designated as days post inoculation (dpi). An overview of the animal experiment is shown in Table 1.

**Table 1:** **Overview of the animal experiment**

**Group no.**	**Animal**	**Sex**	**Age (in months)**	Clinical signs and blood chemistry*	**Gross lesions**	**Liver histopathology**	**Viral antigens in liver by IHC**	
							**Grading**	**Distribution**
**Group 1. Intravenous inoculation of wild boar**	wb93	♀	3	both with reduced feed intake, mild diarrhea, BA, ALT and γGT ↑	in all animals mild hyperplasia of liver lymph nodes and lymphoid tissue in large intestine	both with panlobular hepatocellular swelling, vacuolation and single cell necrosis of hepatocytes	+++	diffuse mainly in Kupffer cells and LSEC
	wb95	♀	3				++	
	wb10	♂	6	both with γGT ↑		both with multifocal lymphoplasmacytic infiltrates and hepatocellular degeneration (mainly centrilobular)	+++	mainly centrilobular in Kupffer cells associated with degenerated hepatocytes
	wb11	♀	6				+++	
**Group 2. Intravenous inoculation of miniature pigs**	mp30**	♀	3	γGT ↑ (not in mp30)	nematodes in gut and milk spots in liver (mp39)	multifocal lymphoplasmacytic infiltrates and single cell necrosis of hepatocytes (mp37 and mp39)	0	diffuse mainly in Kupffer cells and LSEC
	mp37	♀	3				+	
	mp39	♀	3				++	
	mp40	♀	3				0	
**Group 3. Contact infection of wild boar and miniature pigs**	wb87	♂	3	reduced feed intake, mild diarrhea, BA, ALT and γGT ↑	mild hyperplasia of liver and intestinal lymph nodes, altered liver consistency and milk spots, moderate splenomegaly	intralobular lymphohistiocytic infiltrates and single cell necrosis of hepatocytes	++	mainly centrilobular in Kupffer cells and LSEC
	mp63	♀	3	all with γGT ↑	mild hyperplasia of lymphoid tissue in large intestine, nematodes in gut and renal cyst (mp68)	all with mild lymphohistiocytic infiltrates within liver lobules	0	-
	mp68	♂	3				0	
	mp79	♀	3				0	

**Figure 1:**
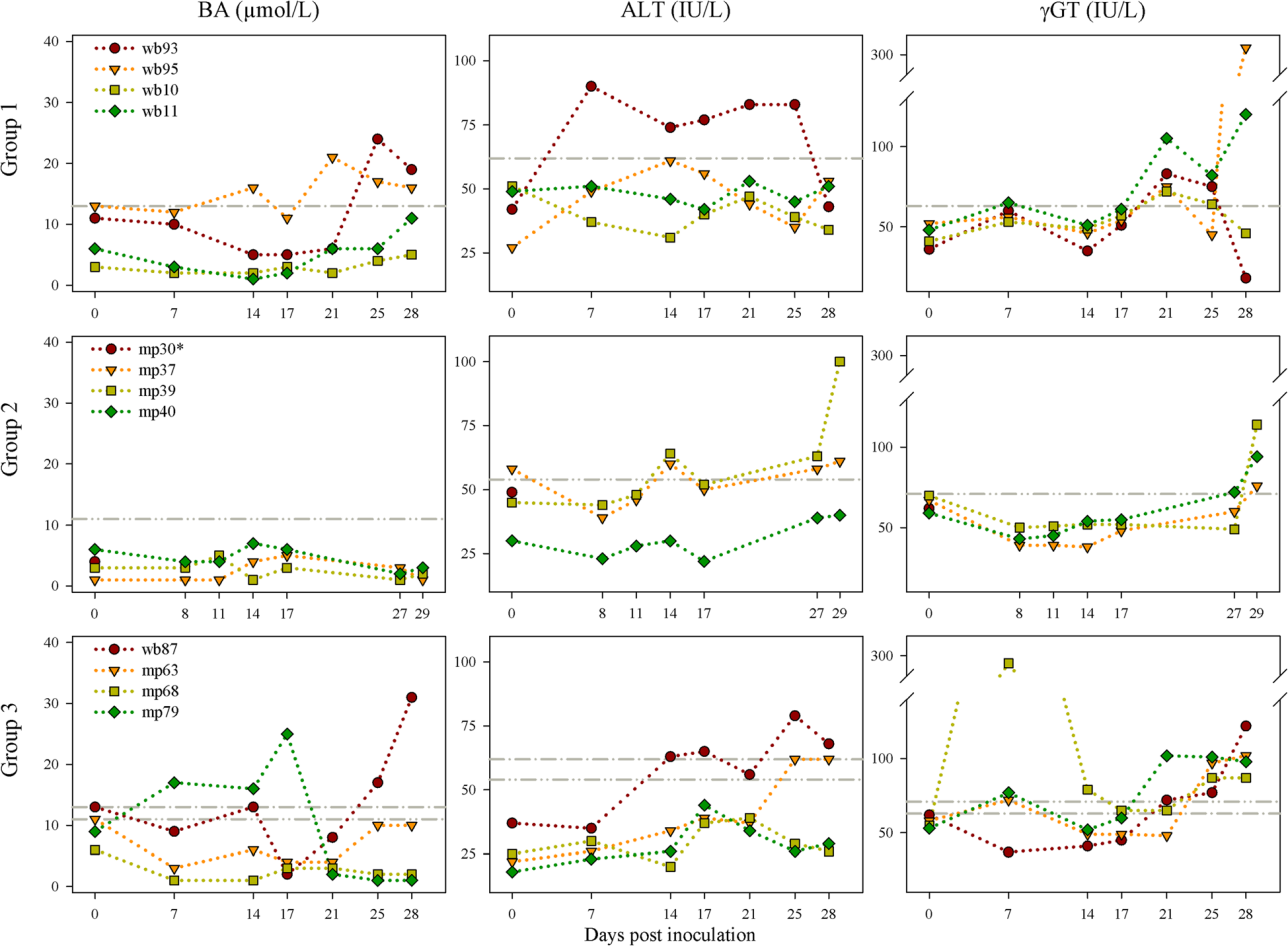
**Detection of bile acids (BA), alanine aminotransferase (ALT) and gamma-glutamyl transferase (γGT) in serum.** Group 1: Intravenous inoculation of wild boar. Group 2: Intravenous inoculation of miniature pigs. Group 3: Contact infection of wild boar and miniature pigs. For the evaluation of the results, upper reference value ranges for the tested biochemical parameters were calculated. Therefore, different serum samples of the negative control wild boar and miniature pigs were analysed (for each subspecies *n* =13). Upper reference range limit for wild boar = grey dot-dashed line. Upper reference range limit for miniature pig = grey dot-dot-dashed line. IU = international units. * Sudden death at 1 dpi (after blood collection).

Tissues were taken on days 29 (Group 2) and 28 (Group 1, Group 3). Grades are formulated on a result of viral antigen density throughout a uniform tissue type. Sections were graded on two separate occasions, without referring to previous recorded results to help standardize the classification. Definition of immunolabelling grades as: 0 = no antigen staining seen, + = mild immunolabelling, ++ = moderate antigen staining, +++ = marked immunolabelling. LSEC = liver sinusoidal endothelial cells. *For details see Figure 1. **Sudden death at 1 dpi (after blood collection). ↑ = elevated biochemical parameter.

Measurements of the body weight and rectal temperature as well as collection of blood and faecal samples were done at time points 0, 1, 3, 5, 8, 11, 14, 17, 21, 24, 27, 29 dpi in Group 2 and 0, 3, 7, 10, 14, 17, 21, 25, 28 dpi in Group 1 and 3. Fever was defined as a body temperature ≥40.0 °C for at least two consecutive days. Aliquots of serum samples were stored at −20 °C for antibody detection and clinical chemistry, and at −70 °C for RNA extraction. Faecal samples were diluted in isotonic saline solution (10%, w/v) and stored at −70 °C for RNA extraction. The experiment was finished after 29 dpi (Group 2) or after 28 dpi (Group 1 and 3). At necropsy, tissue samples (liver, liver lymph node, mesenteric and mandibular lymph nodes, gall bladder, small and large intestine, pancreas, kidney, spleen, tonsil, heart, brain, gonads, uterus or prostate, and quadriceps femoris muscle) were collected for virological, histopathological and immunohistochemical investigations. One part of each tissue sample was fixed immediately in 4% neutral buffered formalin for histological examination and the other part was stored at −70 °C for RNA extraction.

The experiments were approved by the competent authority of the Federal State of Mecklenburg-Western Pomerania, Germany, on the basis of national and European legislation, namely the EU council directive 86/609/EEC for the protection of animals used for experiments (LALLF M-V/TSD/7221.3-2.1.-014/10).

### Clinical chemistry

Serum samples were analysed longitudinally by a spectrophotometric method in an automated analyzer (VetScan Chemistry Analyzer, Abaxis, Union City, USA) using special rotors (VetScan Mammalian Liver Profile reagent rotor, Abaxis) to provide quantitative determinations for alanine aminotransferase (ALT), albumin (ALB), alkaline phosphatase (ALP), bile acids (BA), total bilirubin (TBIL), total cholesterol (CHOL), gamma-glutamyl transferase (γGT) and blood urea nitrogen (BUN) in serum. For the evaluation of the results, upper reference value ranges for the tested biochemical parameters were calculated. Therefore, different serum samples of the negative control wild boar and miniature pigs were analysed (for each subspecies *n* =13).

### Antibody and RNA detection

Sera were tested for the presence of total anti-HEV antibodies with a species independent HEV-Ab ELISA kit (Axiom, Bürstadt, Germany) according to the manufacturer’s instructions. The ELISA uses recombinant HEV gt1 antigens for the detection of anti-HEV antibodies in serum or plasma. Values of the optical density at 450 nm (OD450) equal to or greater than 1 are prescribed as seropositive.

Manual extraction of viral RNA from all serum samples and faecal suspensions was performed using the QIAamp® Viral RNA Mini Kit (QIAGEN GmbH, Hilden, Germany) according to manufacturer’s recommendations. From all tissue samples, viral RNA was extracted using the RNeasy Mini Kit (QIAGEN GmbH). For both extraction methods, an internal control RNA (IC2) was added as described previously [43]. HEV RNA was detected by a novel diagnostic quantitative real-time RT-PCR assay (RT-qPCR) using the CFX96™ Real-Time System (Bio-Rad Laboratories GmbH, München, Germany). All primer and probes used in this study are listed in Table 2. The RT-qPCR was performed using the QuantiTec Probe RT-PCR kit (QIAGEN GmbH) in 25 μL reaction volume with final concentrations of each primer with 0.8 μM, and of the probe with 0.1 μM. A volume of 5 μL RNA was added. Reverse transcription (RT) was carried out at 50 °C for 30 min, followed by denaturation/activation at 95 °C for 15 min. DNA was amplified immediately with 45 cycles at 95 °C (10 s), 55 °C (25 s) and 72 °C (25 s). The determination of the HEV copy number was carried out using a standard curve according to a synthetic external calibrator encompassing the 81 bp sequence of the RT-qPCR amplicon.

**Table 2:** **Primers and probe used in this study**

**Region**	**Primers and probe**	**Position**	**Sequence**	**Product length**
ORF 3	Forward primer1 (HEV.Fa)	5278-5294	GTGCCGGCGGTGGTTTC	81 bp
	Forward primer2 (HEV.Fb)	5278-5296	GTGCCGGCGGTGGTTTCTG	
	Reverse primer (HEV.R)	5340-5359	GCGAAGGGGTTGGTTGGATG	
	Probe (HEV.P)	5300-5320	FAM-TGACMGGGTTGATTCTCAGCC-BHQ1	

ORF = open reading frame.

### Histopathology and immunohistochemistry

For histopathological examinations formalin fixed tissue samples were stained with hematoxylin and eosin (HE) according to standard protocols. For immunohistochemistry (IHC) 3 μm sections were cut, deparaffinised and rehydrated. The pretreatment included a blocking step for the endogenous peroxidase using 3% H_2_O_2_/methanol for 30 min, followed by an antigen retrieval step in the microwave for 10 min at 600 W. A 1:200 diluted commercially available polyclonal rabbit anti-human CD3 antibody (Dako Deutschland GmbH, Hamburg, Germany), which is also binding to porcine CD3 antigen, was used to characterise the inflammatory response in the liver. Viral antigens were detected using a rabbit anti-HEV gt3 hyperimmune serum (rHEVgt3-HIS) in a 1:1000 dilution. For the production of this serum, a rabbit was immunised with an *Escherichia coli* expressed and purified His-tagged C-terminal segment of HEV gt3 capsid protein [21]. The slides were incubated with biotinylated goat anti-rabbit immunoglobulin (Vector Laboratories, LINARIS, Dossenheim, Germany) and an avidin/biotinylated enzyme complex (VECTASTAIN®ABC Reagent, Vector Laboratories, Burlingame, United States of America) followed by visualisation with 3,3-Diaminobenzidine (DAB, Sigma-Aldrich Chemie GmbH, Steinheim, Germany). The viral antigen density was graded as follows: 0 = no antigen staining seen; + = mild immunolabelling (<20% positive cells); ++ = moderate antigen staining (20 – 40% positive cells); +++ = marked immunolabelling (>40% positive cells). The sections were examined independently on two separate occasions.

## Results

Experiments were carried out to determine i.) the pathogenesis of wild boar-derived HEV gt3 in wild boar and miniature pigs (Groups 1 and 2), and ii.) the horizontal transmissibility following such infections (Group 3).

i.) Intravenous inoculation of wild boar (Group 1) and miniature pigs (Group 2) with HEV gt3 of wild boar origin:

### Clinical and biochemical parameters

Intravenously inoculated wild boar did not develop a significant rise in their body temperatures. Only wb93 and wb95 developed moderate clinical signs such as mild depression, slight diarrhea and mild anorexia, and displayed an increase in the BA and γGT serum levels. An elevated ALT level was only observed in wb93. Increase of γGT levels were observed in all intravenously infected wild boar after 21 dpi. Interestingly, ALT and γGT serum levels returned to normal in wb93 after day 25. None of the intravenously inoculated miniature pigs showed febrile temperatures or any other clinical signs, and none of these animals had altered BA serum levels. Increased γGT levels were observed in mp39 and mp40 at 29 dpi, whereas an elevation of the ALT level was only seen in mp39. An overview of the clinical signs is given in Table 1. Figure 1 depicts the time line of serum levels of BA, ALT and γGT. Other biochemical parameters remained within normal limits (data not shown).

### Serology and HEV RNA detection

**Figure 2:**
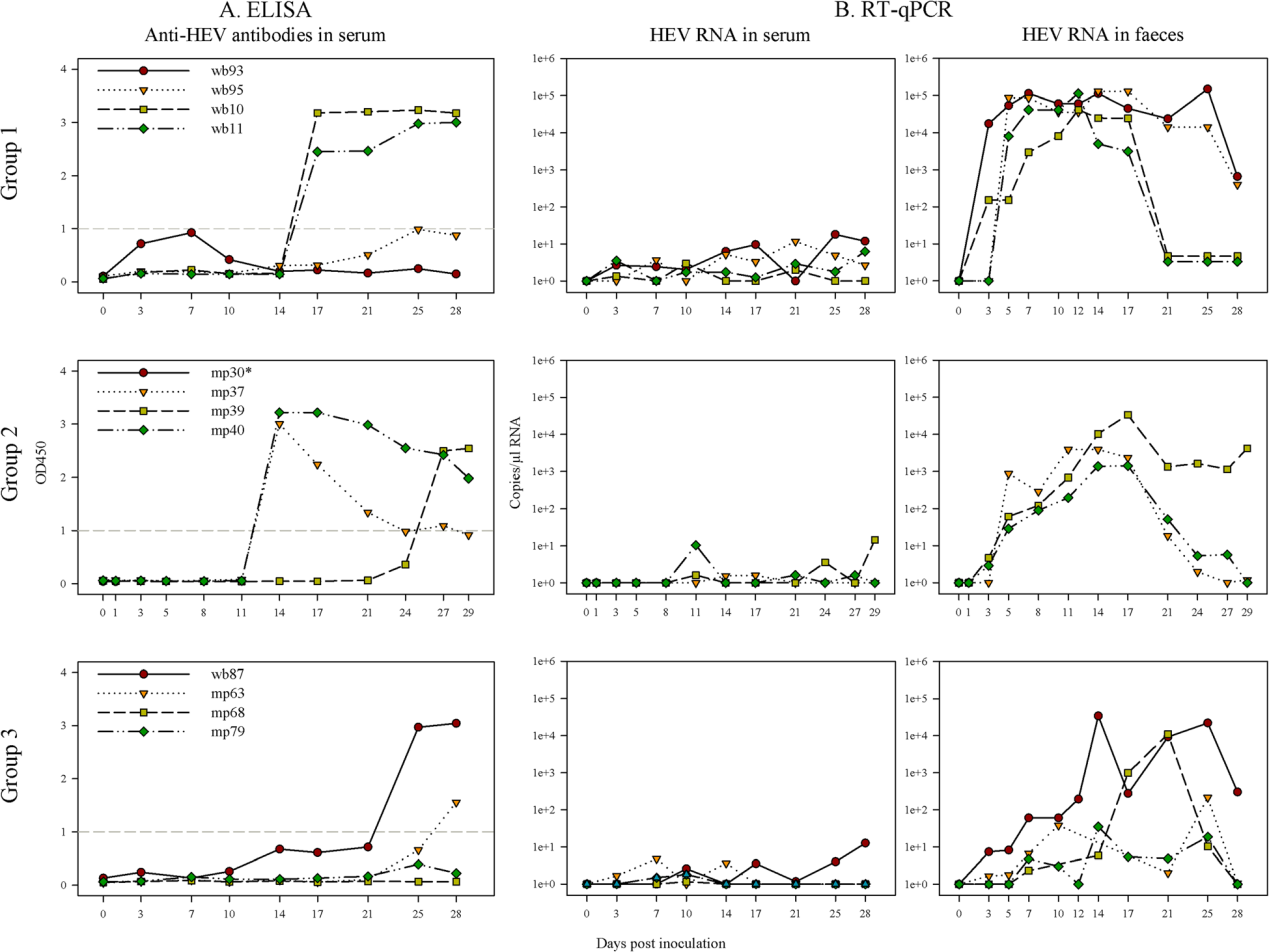
**Serology and HEV RNA detection.** Group 1: Intravenous inoculation of wild boar. Group 2: Intravenous inoculation of miniature pigs. Group 3: Contact infection of wild boar and miniature pigs. **A)** Antibody responses to HEV in serum of inoculated wild boar and miniature pigs measured by a double-antigen sandwich ELISA. OD450-values ≥1 are prescribed as seropositive – this threshold is indicated as a grey-dashed line. **B)** HEV RNA in serum and faeces of HEV inoculated wild boar and miniature pigs estimated by RT-qPCR. * Sudden death at 1 dpi (after blood collection).

Two of the four intravenously inoculated wild boar (wb10 and wb11) seroconverted after 17 dpi, whereas the other two (wb93 and wb93) showed no seroconversion during the observation period (28 days). In the intravenously inoculated miniature pigs antibodies were detected first time in mp37 and mp40 at 14 dpi, whereas mp39 seroconverted thirteen days later. The serological results regarding HEV antibodies are shown in Figure 2A. HEV RNA with copy numbers exceeding 10 copies per μL RNA was found in the sera of two intravenously inoculated wild boar (wb93 at 17, 25, 28 dpi and wb95 at 21 dpi). Viral RNA was detectable in faeces of all wild boar within 3 to 5 dpi at levels of up to 10^5^ copies per μL RNA (wb93, wb95 and wb11). In the two wild boar without seroconversion (wb93 and wb95), RNA copy numbers in faeces persisted until to the end of the experiment, albeit at slightly reduced levels. The two animals which seroconverted (wb10 and wb11) showed a marked reduction of faecal HEV shedding (< 10^1^ copies per μL RNA) eventually. Viral RNAs were detected in liver, gall bladder, caecum, colon and spleen of all intravenously inoculated wild boar. In wb95 positive signals (>20 copies per μL RNA) were also found in brain, muscle and uterus. In general, comparable RNA copy numbers in serum and faeces were found in the miniature pigs. A marked decrease of faecal HEV shedding was also observed in two miniature pigs which seroconverted within 14 dpi (mp37 and mp40). In most of the intravenously infected miniature pigs viral RNAs were also detected in liver, gall bladder, caecum, colon and spleen. Already at 1 dpi, relatively high viral RNA copy numbers were found in the liver of mp30. The results of HEV RNA in serum and faeces are shown in Figure 2B. Table 3 summarises the viral loads of selected tissue samples. Additionally, HEV RNA was found in bile of wb93, wb95, wb11 and mp39.

**Table 3:** **Results of RT-qPCR analysis of selected tissue samples from wild boar (wb) and miniature pigs (mp)**

**Tissue**	**CT-value**	**Goup 1. Intravenous inoculation of wb**				**Group 2. Intravenous inoculation of mp**				**Group 3. Contact infection of wb and mp**			
	***copies/μL RNA***	**wb93**	**wb95**	**wb10**	**wb11**	mp30*	**mp37**	**mp39**	**mp40**	**wb87**	**mp63**	**mp68**	**mp79**
**Liver**		20.7	22.6	24.1	24.6	28.1	32.3	24.3	No CT	23.0	24.2	No CT	35.3
		*6521.0*	*2259.6*	*952.4*	*685.8*	*94.6*	*8.4*	*834.2*		*1763.9*	*888.8*		*1.5*
**Gall bladder**		24.0	22.0	32.0	31.0	32.5	No CT	27.0	No CT	30.0	No CT	No CT	35.9
		*991.5*	*3137.8*	*9.9*	*17.6*	*7.3*		*175.1*		*31.3*			*1.0*
**Duodenum**		27.6	27.2	31.4	No CT	34.5	No CT	29.1	No CT	31.1	No CT	No CT	No CT
		*124.0*	*157.0*	*13.8*		*2.3*		*51.3*		*16.9*			
**Jejunum**		31.6	24.9	No CT	No CT	34.1	No CT	31.8	No CT	29.6	No CT	No CT	No CT
		*12.3*	*577.0*			*3.0*		*11.0*		*40.3*			
**Ileum**		33.3	30.0	34.4	No CT	35.4	No CT	33.8	No CT	29.9	No CT	No CT	No CT
		*4.8*	*31.7*	*2.6*		*1.4*		*3.5*		*33.1*			
**Caecum**		25.9	32.3	35.3	34.8	35.5	No CT	26.5	No CT	25.0	35.9	No CT	35.0
		*337.7*	*8.4*	*1.5*	*2.0*	*1.3*		*23.,9*		*557.4*	*1.0*		*1.8*
**Colon**		34.4	23.7	31.8	34.4	No CT	No CT	28.9	33.8	25.0	34.2	No CT	No CT
		*2.6*	*1171.8*	*10.8*	*2.4*			*60.7*	*3.6*	*557.4*	*2.7*		
**Rectum**		24.9	30.2	No CT	No CT	N. d.	No CT	32.6	No CT	33.0	No CT	No CT	No CT
		*607.7*	*28.2*					*7.2*		*5.5*			
**Pancreas**		No CT	34.7	No CT	No CT	N. d.	No CT	No CT	No CT	No CT	No CT	No CT	No CT
			*2.1*										
**Mandibular LN**		No CT	No CT	No CT	33.6	No CT	No CT	No CT	No CT	No CT	No CT	No CT	No CT
					*3.9*								
**Kidney**		34.7	33.2	No CT	No CT	No CT	No CT	31.7	No CT	No CT	No CT	No CT	No CT
		*2.0*	*5.0*					*11.6*					
**Spleen**		31.0	31.7	34.1	34.0	35.8	No CT	32.5	No CT	34.0	No CT	No CT	No CT
		*17.6*	*11.7*	*3.0*	*3.1*	*1.1*		*7.4*		*3.1*			
**Heart**		No CT	33.9	32.6	No CT	N. d.	No CT	No CT	No CT	34.6	No CT	No CT	No CT
			*3.3*	*6.9*						*2.2*			
**Brain**		32.3	28.9	No CT	No CT	N. d.	No CT	No CT	No CT	No CT	No CT	No CT	No CT
		*8.2*	*59.3*										
**Muscle**		No CT	30.7	No CT	No CT	No CT	No CT	No CT	No CT	No CT	No CT	No CT	No CT
			*20.7*										
**Ovary/Testicle**		No CT	30.9	No CT	No CT	N. d.	No CT	No CT	No CT	No CT	No CT	No CT	No CT
			*19.1*										
**Uterus/Prostate**		No CT	30.6	No CT	No CT	N. d.	No CT	35.0	No CT	No CT	No CT	No CT	No CT
			*22.3*					*1.8*					

Tissues were taken on days 29 (Group 2) and 28 (Group 1, Group 3). No CT ≥36.0 (= negative). Viral copy numbers in tissues were calculated from CT values determined by RT-qPCR. LN = lymph node. N. d. = not determined. *Sudden death at 1 dpi (after blood collection).

### Pathology

**Figure 3:**
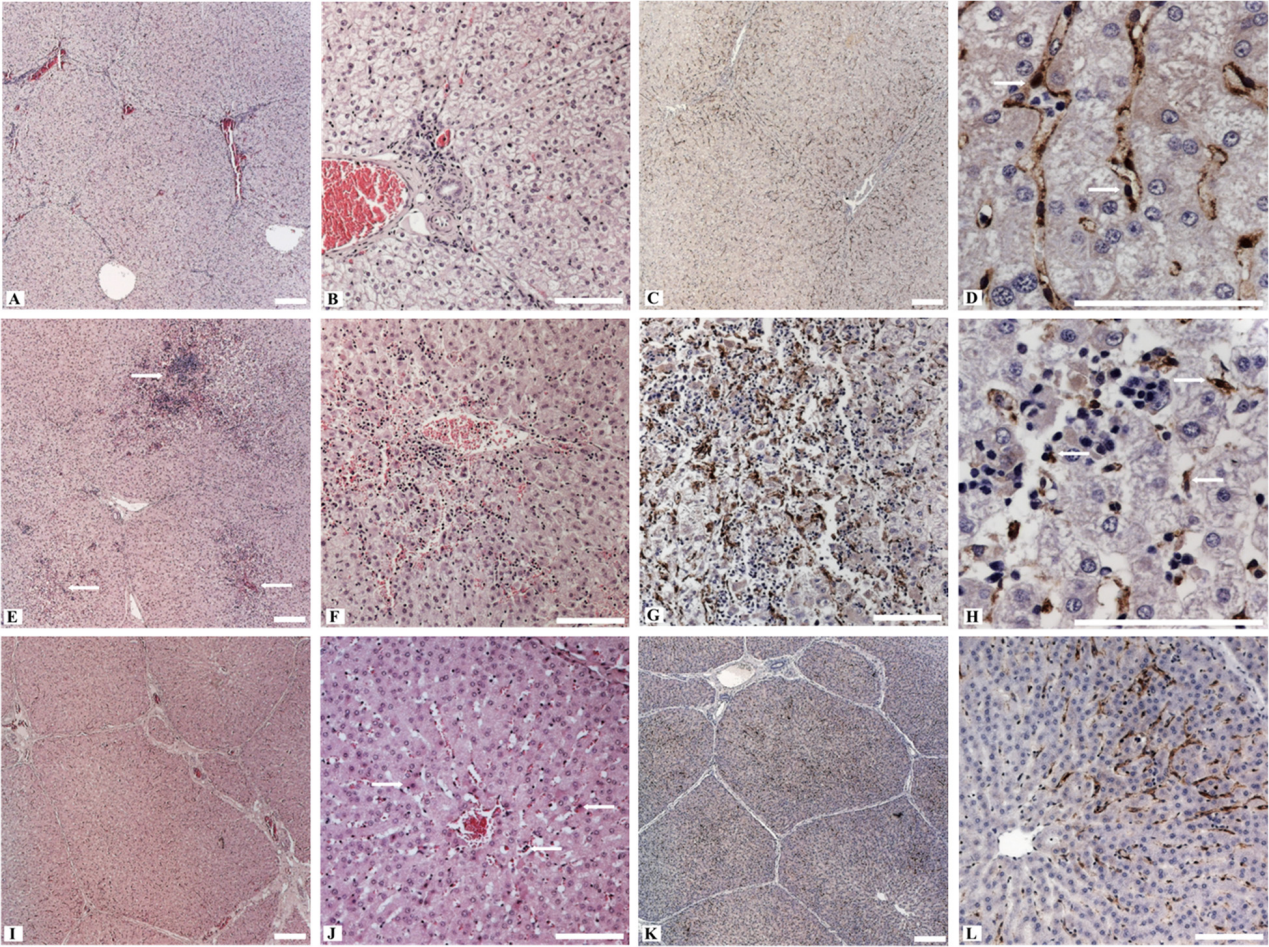
**Histopathological alterations and immunohistochemistry of the liver from intravenously infected wild boar (Group 1) and miniature pigs (Group 2). A)** Hepatic lobules with moderate hyperaemia of sinusoids and portal fields (wb95). **B)** The lobules show swelling and vacuolation of hepatocytes (wb95). **C)** Diffuse distribution of viral antigens within the liver lobules (wb95). **D)** Marked immunolabelling within a hepatic lobule, intracytoplasmatic mainly in Kupffer cells (arrows) and liver sinusoidal endothelial cells (wb93). **E)** Multifocal hepatocellular degeneration with focus on centrilobular areas (arrows) and hyperaemic central veins (wb11). **F)** Centrilobular area of hepatocellular degeneration (apoptotic bodies) with infiltrates of lymphocytes, plasma cells and Kupffer cells (wb10). **G)** Viral antigens within the centrilobular area of a liver lobule in association with degenerated hepatocytes and inflammatory infiltrates (wb11). **H)** Viral antigens within an area of hepatocellular degeneration, mainly in association with Kupffer cells (arrows) and some hepatocytes (wb10). **I)** Hepatic lobule with mild hyperaemia of sinusoids and portal fields (mp39). **J)** Areas of spotty necrosis and apoptotic bodies (arrows) with slight infiltrates of lymphocytes and Kupffer cells (mp37). **K)** Diffuse distribution of viral antigens within the liver lobules (mp39). **L)** Intense immunolabelling within a hepatic lobule, mainly in association with Kupffer cells and liver sinusoidal endothelial cells (mp39). All scale bars represent 100 μm.

**Figure 4:**
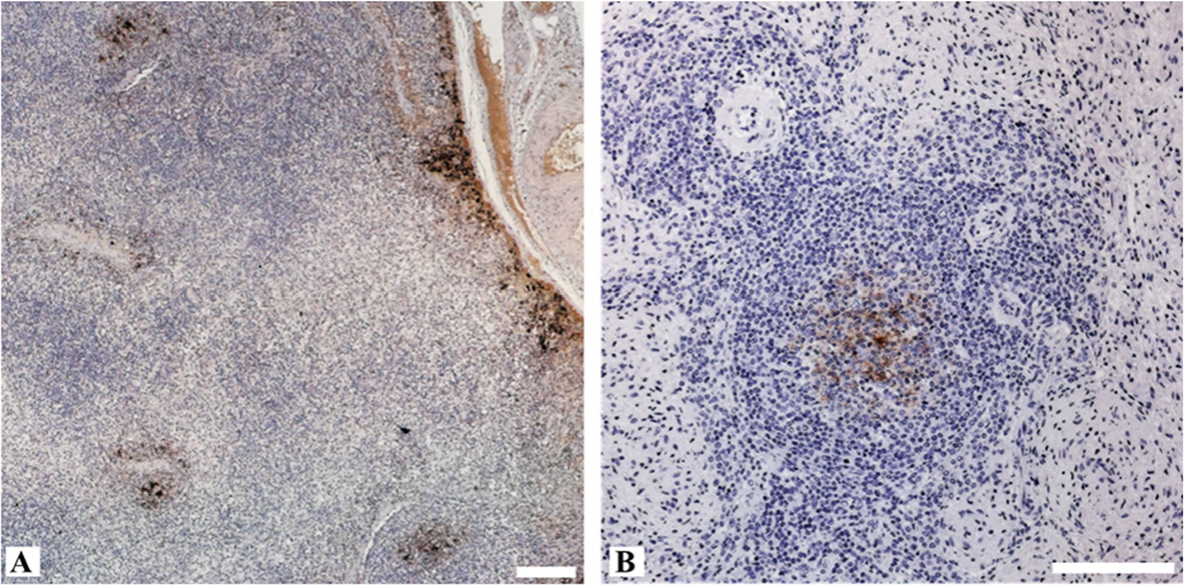
**Immunohistochemistry of liver lymph node and spleen from intravenously HEV inoculated wild boar (Group 1). A)** Viral antigens in the subcapsular layer and in the germinal centre of secondary follicles of a liver lymph node (wb93). **B)** Splenic immunolabelling of viral antigens in the germinal centre of a lymphoid follicle (wb11). All scale bars represent 100 μm.

No gross pathological changes specific for viral hepatitis were seen in the livers of any of the pigs. However, macroscopic signs like mild to moderate follicular hyperplasia of lymphoid tissues in large intestine and mild hyperplasia of liver lymph nodes in intravenously inoculated wild boar were seen in some animals. Necropsy revealed also moderate intestinal nematode infestations and multifocal white spots (“milk spots”) in the liver characteristic for chronic infection with Ascaris suum. Intralobular lesions of different sizes, patterns and frequencies associated with varying distribution of viral antigens were observed in the liver of intravenously inoculated pigs, but not in the negative control animals. Mild to moderate periportal lymphoplasmacytic infiltrates and Kupffer cell proliferations were also found in the liver of all intravenously inoculated pigs. However, no viral antigens were detected in periportal fields. By immunohistochemistry, viral antigens within hepatic lobules were found mainly in Kupffer cells and liver sinusoidal endothelial cells, and to a lesser extent in hepatocytes. Partially, hepatic lesions and infiltrates of CD3-positive cells were associated with viral antigens in Kupffer cells and liver sinusoidal endothelial cells. In detail, the liver of intravenously inoculated wild boar showed a more heterogeneous pattern in comparison to the miniature pigs. Two animals (wb93 and wb95) revealed a mild to moderate multifocal intralobular lymphohistiocytic infiltration with multifocal hepatocellular swelling and vacuolation, and a diffuse viral antigen distribution (Figure 3A-D). However, more severe liver lesions were observed in the two other intravenously inoculated wild boar (wb10 and wb11), with a pronounced mainly centrilobular hepatocellular degeneration associated with infiltrates of lymphocytes, plasma cells and Kupffer cells (Figure 3E and F). These lesions were clearly associated with a marked immunolabelling of viral antigens (Figure 3G and H). The liver of intravenously inoculated miniature pigs displayed diffuse mild intralobular lymphoplasmacytic infiltrations with single cell necrosis of hepatocytes and small to moderate amounts of diffusely distributed viral antigens (Figure 3I-L). In some animals, viral antigens were also found in the sub-capsular layer and follicles of distinct lymph nodes and in the germinal centre of lymphoid follicles in spleen (Figure 4A and B). In none of the other tested tissues viral antigens were found. An overview of gross lesions, histopathology and immunohistochemistry is given in Table 1. All IHC results of viral antigen detection are summarised in the Additional file 1.

ii.) Transmission of HEV gt3 from intravenously inoculated wild boar to contact wild boar and miniature pigs (Group 3).

### Clinical and biochemical parameters

None of the contact animals became pyrexic. Only wb87 showed moderate clinical signs characterized by mild depression, slight diarrhea and reduced feed intake in combination with elevated BA and ALT serum levels after 25 dpi. In the contact miniature pig mp79 a slight increase of BA in serum was also observed (7 to 17 dpi). All contact animals had increased γGT serum levels at different time points, most strikingly in mp68 at 7 dpi. An overview of the clinical signs is given in Table 1. Figure 1 depicts the time lines of serum levels of BA, ALT and γGT. Other tested biochemical parameters remained within normal limits (data not shown).

### Serology and HEV RNA detection

Two out of four contact pigs seroconverted during the observation period (wb87 at 25 dpi and mp63 at 28 dpi), while the other two contact animals (mp68 and mp79) developed no detectable HEV antibodies during this time. Serological results are shown in Figure 2A. Only wb87 was tested positive for viral RNA in serum with over 10 copies per μL RNA at 28 dpi. Viral RNA in faecal samples was detectable in all contact pigs with over 10 copies per μL RNA after 7 dpi in wb87, and after 10 to 17 dpi in the miniature pigs. The results of HEV RNA in serum and faeces are shown in Figure 2B. Viral RNA was detected in liver, gall bladder, small and large intestine, and spleen of wb87. The livers of the contact miniature pigs were tested positive for HEV RNA as well, except for mp68. Compared to the contact miniature pigs, higher viral copy numbers were detectable in wb87. Table 3 summarises the viral loads of selected tissue samples. Viral RNA was also found in bile of mp163 and wb87.

### Pathology

**Figure 5:**
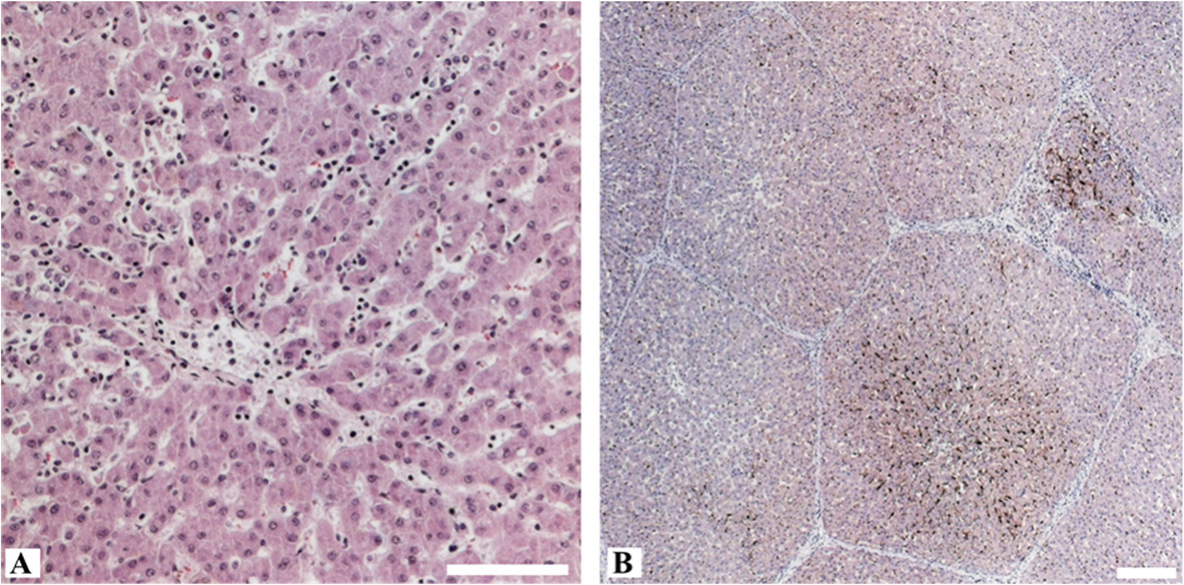
**Histopathological alterations and immunohistochemistry of the liver from the contact wild boar (Group 3). A)** Intralobular area with inflammatory infiltrates mostly lymphocytes and histiocytes. **B)** Multifocal distribution of viral antigens especially in centrilobular areas (arrows). All scale bars represent 100 μm.

No gross pathological changes specific for viral hepatitis were seen in the livers of any of the contact animals, but a mild follicular hyperplasia of lymphoid tissues in intestine and of liver lymph nodes were frequently seen. Additionally, moderate splenomegaly was found in the infected contact wild boar. All contact animals showed a randomly distributed multifocal mild lymphohistiocytic infiltration in the liver associated with single cell necrosis (Figure 5A). However, viral antigens were only found in the liver of the contact wild boar (Figure 5B). In the miniature pigs, viral antigens were demonstrated exclusively in the follicles of the mandibular lymph nodes. An overview of gross lesions, histopathology and immunohistochemistry is given in Table 1. All IHC results of viral antigen detection are summarised in the Additional file 1.

## Discussion

To date, the food-borne zoonotic transmission of HEV gt3 in Europe is primarily associated with domestic pigs [4], while data on the pathogenicity of HEV in wild boar and their role in HEV transmission to domestic pigs are missing. HEV prevalence studies in hunted wild boar and serological studies in humans being in contact with them suggest zoonotic transmissions [21,22,27,29-31]. Several HEV transmission studies in domestic pigs were performed [35,36,38,40], but none involving wild boar. Therefore, the current study was carried out to elucidate the transmission and pathogenesis of wild boar-derived HEV gt3 in European wild boar and in miniature pigs. Compared to common domesticated swine breeds, the miniature pig offers several breeding and handling advantages. Miniature pigs have been used extensively already in several fields of biomedical research [44], but HEV infection studies have never been carried out in this pig breed.

The experimental inoculation of wild boar and miniature pigs reveals an efficient HEV replication with substantial virus shedding. Following intravenous challenge of wild boar, HEV infection was successfully transmitted to contact animals. These contact animal infections resemble the natural course of the disease. Wild boar-derived HEV gt3 was detected in serum, faeces and different tissues of all intravenously inoculated pigs. Moreover, an early virus replication in the liver was observed in one intravenously infected animal already at 1 dpi. Our findings confirm that the liver is the primary location of HEV replication. Extra-hepatic replication sites have been reported [45] and in this study, HEV RNA or viral antigens were also observed in liver, spleen and different lymph nodes. Interestingly, HEV RNA was detected in the brain of two out of four intravenously inoculated wild boar. Neurotropic HEV gt3 variants in humans are under discussion and HEV RNA was detected recently in the cerebrospinal fluid of patients with chronic HEV infection and neurological symptoms [46]. Of course a contamination of tissue specimens with HEV containing blood cannot be excluded completely, but is rather unlikely as the viral loads of all collected serum samples were mostly below those detected in different tissue samples. The duration of faecal HEV shedding in most intravenously inoculated pigs was similar, but higher viral loads were found in faeces of wild boar. In intravenously inoculated animals anti-HEV antibodies were first time detected 14 dpi which is in line with results obtained in previous studies [37,47]. However, seroconversion between three and eight weeks post infection were more often reported [40,48]. The ELISA system used in this study detects all classes of antibodies to HEV in serum. Therefore, the rise of antibody titres observed in the current experiment cannot be associated with a single Ig class. HEV total antibody levels might be also influenced by HEV-specific immunoglobulin (Ig) A in serum, as IgA can be detected in serum of patients with hepatitis E [49,50]. Moreover, a reduction of virus shedding in faeces was observed in the intravenously inoculated pigs which seroconverted after 14 to 17 dpi. Our findings support the hypothesis that adaptive immune responses are crucial to control HEV infection [51].

The faecal-oral transmission of HEV is considered to be the main transmission route among pigs [48]. Based on a study in non-human primates, the infectious dose of HEV required for oral infection is assumed to be higher than for intravenous infection [52]. In our study, the infectious dose of the contact animals remains unknown. It can be assumed that the contact wild boar might have had a higher exposure to HEV because of their direct and permanent contact to excreta of the infected animals than the miniature pigs which were only exposed to collected faeces. As HEV RNA was detected in urine of experimentally infected domestic pigs [35], HEV might be also transmitted via urine. The reason for the lacking antibody response in two contact miniature pigs, despite elevated enzyme levels and viral shedding via faeces, remains unclear. Most probably the duration of the experiment was not long enough or the HEV infection was not systemic, as described before [39]. In a previous study it could be demonstrated that domestic pigs could be infected orally, nevertheless, not each contact pig was infected and the antibody response was less efficient as compared to the intravenous inoculation route [45].

In former studies only subclinical HEV infections have been described in domestic pigs [35-37]. In the present study a clinical course of HEV infection in pigs could be proven, based on elevated γGT-levels in serum. Increased γGT-levels have also been reported for experimentally HEV-infected non-human primates [53], but not described for pigs before. Moreover, increased ALT and BA serum levels were also observed, especially in wild boar and to a lesser extent in miniature pigs. In the infected wild boar piglets the moderate clinical signs were concomitant with increasing serum levels of BA and liver enzymes. Obviously these results support laboratory findings in humans with HEV infection which are similar to other forms of viral hepatitis and characterised also by elevated serum levels of ALT and γGT [54] due to marked hepatic necrosis and cellular exhaustion of enzymes [55]. Also elevated levels of BA in serum are found in viral hepatitis in humans [56]. The inter-individual variations in these parameters may have resulted from factors such as age, sex, physical condition and unrelated co-infections.

Swelling of hepatocytes with vacuolation of the cytoplasm was seen in acute liver injury of domestic pigs as a result of the HEV infection as described before [35]. Non-lipid hepatocellular vacuolation is attributed to alterations in the injured cell as a result of hydropic change, but may also reflect a beneficial cellular adaptation rather than degenerative change [51]. Especially in wild boar mild to moderate intralobular lymphoplasmacytic or lymphohistiocytic infiltrates with variable degree of hepatocellular degeneration were found histopathologically. In previous studies microscopic liver lesions with multifocal lymphoplasmacytic viral hepatitis were observed in both experimentally [35,37] and naturally [42] HEV infected domestic pigs. Our histopathological findings for hepatic lesions in the miniature pigs were comparable, but varied for wild boar ranging from diffuse moderate lesions with swelling, vacuolation and single cell necrosis of hepatocytes and multifocal moderate to severe hepatocellular degenerations.

Only few immunohistochemical studies on HEV infected animals and humans have been published before [57-60]. HEV has been shown to replicate in hepatocytes and in extra-hepatic tissues such as small intestine, colon, spleen, bile duct and lymph nodes [45,61]. By immunohistochemistry, we were able to detect viral antigens mainly in Kupffer cells and liver sinusoidal endothelial cells, partially associated with hepatic lesions and infiltrates of CD3-positive cells. Since Kupffer cells and liver sinusoidal endothelial cells have antigen presenting functions [62], they may also play a role in the host defense mechanisms and immunopathogenesis. Anyhow, a virus proliferation in these cells is possible, if not essential. Lymphatic tissue might also represent extra-hepatic HEV replication sites, as viral antigens were found in spleen, hepatic and mandibular lymph nodes. Interestingly, no viral antigens were detected by immunohistochemistry in intestine as described in a previous HEV infection study in gerbils [63].

Host cell injury in a viral infection may be mediated by either a direct effect of the infectious agent or indirectly through the antiviral host response, or a combination of both. In this study, different patterns in the immunohistochemical detection of HEV antigens and varying lesions were seen in the liver. HEV antigens were either diffusely distributed without association to liver lesions or associated with hepatocellular degeneration, especially in centrilobular areas. As HEV itself appears to be non-cytopathic [64], an immunopathogenesis is assumed for hepatitis E in humans [65]. Previous immunohistochemical studies in liver biopsies of patients with acute hepatitis E revealed that lymphocyte infiltrates consisted mainly of CD3-positive T cells containing a predominantly cytotoxic CD8-positive cell subpopulation which probably is playing an important role in HEV-induced liver injury [66]. Interestingly, CD3-positive T cell infiltrations within liver lesions were also observed in this study. The consistent coincidence of infiltrates of lymphocytes, plasma cells and histiocytes with hepatocellular degenerations and viral antigens supports the assumption that liver damage in pigs might also be immune-mediated.

Two different patterns within the course of HEV infection were observed: Animals with early anti-HEV seroconversion are able to clear the virus, while animals with lacking antibody responses suffered from prolonged HEV persistence until the end of the investigation period. Perhaps a weak cytotoxic response in pigs leads to viral persistence, yet without obvious liver damage, whereas a sufficient immune response may lead to an effective HEV clearance that is accompanied by a variable degree of hepatic damage, however. In humans the course of HEV infection can vary substantially between different individuals and chronic hepatitis E cases have been described in immunosuppressed patients [65]. Recent studies in humans were able to associate the activation of the interferon system and viral evolution with severity or chronicity of hepatitis E [37]. Studies in humans also revealed that chronic hepatitis E might be associated with impaired HEV-specific T-cell responses and enhancing adaptive cellular immunity against HEV might prevent persistent HEV infections [36]. In swine factors like virus titer, ratio of infectious to defective particles, route of infection and host factors like the immune status, age of exposure and the presence of co-infections have been discussed to modulate the clinical outcome of HEV infection [27,28]. In the study presented here, some of the experimental animals were carrying also nematodes and showed mild gastrointestinal symptoms possibly caused by other infectious agents affecting swine, but also by stress or a modified feeding regime. Wild boar piglets used in this study were obtained from a local farmer, therefore pre-existing infections with other pathogens cannot be excluded. Liver homogenates given to the experimental animals were sterile filtered to prevent parasitic and bacterial superinfections. Moreover, clinical and pathological examinations showed no indication of other infections. However, future studies should clarify the impact of co-infections on the HEV pathogenesis. Moreover, the pathomechanisms for the development of persistent HEV infections in pigs should be further assessed as this may provide an animal model for the chronic hepatitis E infection in humans.

Taken together, our data underline the importance of wild boar as reservoir host and for transmission of HEV gt3 to domestic swine and reveal Kupffer cells, liver sinusoidal endothelial cells and extra-hepatic lymphatic cells as potential virus replication sites. Since large amounts of virus particles are excreted in faeces of wild boar, droppings can contaminate the environment and pose a particular risk to susceptible species. Actually, in most industrialised countries the HEV infected population of domestic swine is far larger than those of the wild boar. Accordingly, wild boar and other wildlife also can be at infection risk by using pig manure as fertilizer on agricultural land.

## Additional file

Additional file 1
**HEV-antigen detection within post mortem tissues of wild boar (wb) and miniature pigs (mp) assessed by immunohistochemistry.** All results of viral antigen detection in examined tissues were summarised in this overview. The viral antigen density was graded as follows: 0 = no antigen staining seen; + = mild immunolabelling (<20% positive cells); ++ = moderate antigen staining (20 – 40% positive cells); +++ = marked immunolabelling (>40% positive cells). The sections were examined independently on two separate occasions.
