# Supplementary material for: Natural and experimental hepatitis E virus genotype 3 - infection in European wild boar is transmissible to domestic pigs
Source: Vet Res. 2014 Nov 26;45(1):121. doi: 10.1186/s13567-014-0121-8 (PMC4243386; doi:10.1186/s13567-014-0121-8)
Supplement: Additional file 1 — HEV-antigen detection within post mortem tissues of wild boar (wb) and miniature pigs (mp) assessed by immunohistochemistry. All results of viral antigen detection in examined tissues were summarised in this overview. The viral antigen density was graded as follows: 0 = no antigen staining seen; + = mild immunolabelling (<20% positive cells); ++ = moderate antigen staining (20 – 40% positive cells); +++ = marked immunolabelling (>40% positive cells). The sections were examined independently on two separate occasions. [file 13567_2014_121_MOESM1_ESM.docx]

| **Tissue** | **Group 1. Intravenous inoculation of wb** | | | | **Group 2. Intravenous inoculation of mp** | | | | **Group 3. Contact infection of wb and mp** | | | |
| --- | --- | --- | --- | --- | --- | --- | --- | --- | --- | --- | --- | --- |
|  | **wb93** | **wb95** | **wb10** | **wb11** | **mp30*** | **mp37** | **mp39** | **mp40** | **wb87** | **mp63** | **mp68** | **mp79** |
| **Liver** | +++ ^1^ | ++ ^1^ | +++ ^1^ | +++ ^1^ | 0 | + | ++^1^ | 0 | ++ ^3^ | 0 | 0 | 0 |
| **Liver LN** | + ^2^ | + | + | 0 | N. d. | 0 | + | 0 | 0 | 0 | 0 | 0 |
| **Gall bladder** | 0 | 0 | 0 | 0 | 0 | 0 | 0 | 0 | 0 | 0 | 0 | 0 |
| **Duodenum** | 0 | 0 | 0 | 0 | 0 | 0 | 0 | 0 | 0 | 0 | 0 | 0 |
| **Jejunum** | 0 | 0 | 0 | 0 | 0 | 0 | 0 | 0 | 0 | 0 | 0 | 0 |
| **Ileum** | 0 | 0 | 0 | 0 | 0 | 0 | 0 | 0 | 0 | 0 | 0 | 0 |
| **Caecum** | 0 | 0 | 0 | 0 | 0 | 0 | 0 | 0 | 0 | 0 | 0 | 0 |
| **Colon** | 0 | 0 | 0 | 0 | 0 | 0 | 0 | 0 | 0 | 0 | 0 | 0 |
| **Rectum** | 0 | 0 | 0 | 0 | 0 | 0 | 0 | 0 | 0 | 0 | 0 | 0 |
| **Pancreas** | 0 | 0 | 0 | 0 | N. d. | 0 | 0 | 0 | 0 | 0 | 0 | 0 |
| **Mesenteric LN** | 0 | 0 | 0 | 0 | N. d. | 0 | 0 | 0 | 0 | 0 | 0 | 0 |
| **Mandibular LN** | +++ | 0 | 0 | 0 | N. d. | 0 | 0 | 0 | 0 | 0 | + | + |
| **Kidney** | 0 | 0 | 0 | 0 | 0 | 0 | 0 | 0 | 0 | 0 | 0 | 0 |
| **Spleen** | 0 | 0 | 0 | + ^2^ | 0 | 0 | + | 0 | 0 | 0 | 0 | 0 |
| **Heart** | 0 | 0 | 0 | 0 | 0 | 0 | 0 | 0 | 0 | 0 | 0 | 0 |
| **Muscle** | 0 | 0 | 0 | 0 | 0 | 0 | 0 | 0 | 0 | 0 | 0 | 0 |
| **Lung** | 0 | 0 | 0 | 0 | 0 | 0 | 0 | 0 | 0 | 0 | 0 | 0 |
| Tissues were taken on days 29 (Group 2) and 28 (Group 1, Group 3). Grades are formulated on a result of viral antigen density throughout a uniform tissue type. Masked sections were graded on two separate occasions, without referring to previous recorded results to help standardize the classification. Definition of immunolabelling grades as: 0 = no antigen staining seen, + = mild immunolabelling, ++ = moderate antigen staining, +++ = marked immunolabelling. N. d. = not determined. LN = lymph node. *Sudden death at 1 dpi (after blood collection). ^1^ For details see Figure 3. ^2^ For details see Figure 4. ^3^ For details see Figure 5. | | | | | | | | | | | | |

**Additional file 1 HEV-antigen detection within *post mortem* tissues of wild boar (wb) and miniature pigs (mp) assessed by immunohistochemistry.**
